# Long-term efficacy and safety of low-dose ritonavir-boosted atazanavir (ATV/r) 200/100 mg in HIV-infected Thai patients

**DOI:** 10.1186/1758-2652-13-S4-P60

**Published:** 2010-11-08

**Authors:** A Avihingsanon, T Apornpong, SJ Kerr, J Ananworanich, K Ruxrungtham

**Affiliations:** 1 HIV Netherlands Australia Thailand (HIV-NAT) Rese, Thai Red Cross AIDS Research centre, Bangkok, Thailand; 2 National Centre in HIV Epidemiology and Clinical, The University of New South Wales, Sydney, Australia; 3 South East Asia Research Collaboration with Hawaii, Bangkok, Thailand; 4 Faculty of Medicine, Chulalongkorn University, Bangkok, Thailand

## Purpose of the study

Atazanavir is a good but expensive regimen in resource limited settings (RLS). A previous HIV-NAT study showed that a low dose of ATV/r 200/100mg once daily (QD) provided adequate atazanavir plasma concentrations in HIV-infected Thai adults. Reducing the dose would make atazanavir more accessible to those patients in need in RLS. We aimed to evaluate long term efficacy of lower dose ATV/r 200/100 mg in HIV-infected Thai adults.

## Methods

HIV infected patients commencing ATV/r 200/100 mg QD in a prospective long term cohort at HIV-NAT, Bangkok, Thailand were analysed. CD4, HIV RNA, and safety parameters are performed every 6 months as part of the cohort.

## Summary of results

A total of 84 (51% men) subjects with median age of 40 years and median body weight of 57.6 kg were included in this analysis. The median time of taking lower dose was 63 (IQR 39-82, range 2-237) weeks. 11/84 subjects had HIV RNA > 50 copies/mL at time of ATV/r lower dose initiation. These patients mainly were NNRTI based treatment failure. NNRTI based treatment failure. 79% used tenofovir as backbone. High ATV Ctrough (32%), hyperbilirubinemia (32%), and clinically jaundice (22%) were the main reason for using the lower dose. At time of analysis, 72/74 (97%) patients with availability of at least 1 HIV RNA at 6 months interval, had HIV RNA <50 copies/mL. The median CD4 count was significantly increased from 394 to 456 cells/mm^3^ (P =0.010). The median change in fasting cholesterol, triglyceride, LDL and HDL were -6 , -11, 13.8 and 4 mg/dL, respectively. However, the changes were not statistically significant exception for HDL (p=0.006). In patients previously used standard dose of ATV/r 300/100 QD, the median of bilirubin was 1.9 (range 0.1-7.7) mg/dl and it improved significantly after dose reduction (p = 0.003). Majority of the patients well tolerated to the treatment, only 9 patients, ATV/r 200/100 mg QD was discontinued due to ran out of stock (4 patients), hepatitis (2 patients), hyperbilirubinemia (1), nephrotic syndrome (1), and non-adherence (1). ATV Ctrough concentrations of ATV/r 200/100 were available in only 51 cases, all had ATV Ctrough concentrations >0.15mg/L.

## Conclusions

Regimens with ATV/r 200mg/100mg provided adequate ATV plasma concentrations, were effective and well tolerated in HIV-infected Thai adult patients. A randomized controlled trial should be conducted to confirm our findings. This data will be benefit to a million of HIV infected in RLS.
